# Non-inferiority versus superiority drug claims: the (not so) subtle distinction

**DOI:** 10.1186/s13063-017-2024-2

**Published:** 2017-06-15

**Authors:** Jitendra Ganju, Dror Rom

**Affiliations:** 1 Global Blood Therapeutics, South San Francisco, CA 94080 USA; 2 Prosoft Clinical, Wayne, USA

**Keywords:** Non-inferiority, Superiority, Margin, Confidence interval, Trial design

## Abstract

**Background:**

Current regulatory guidance and practice of non-inferiority trials are asymmetric in favor of the test treatment (Test) over the reference treatment (Control). These trials are designed to compare the relative efficacy of Test to Control by reference to a clinically important margin, *M*.

**Main text:**

Non-inferiority trials allow for the conclusion of: (a) non-inferiority of Test to Control if Test is slightly worse than Control but by no more than *M*; and (b) superiority if Test is slightly better than Control even if it is by less than *M*. From Control’s perspective, (b) should lead to a conclusion of non-inferiority of Control to Test. The logical interpretation ought to be that, while Test is statistically better, it is not clinically superior to Control (since Control should be able to claim non-inferiority to Test). This article makes a distinction between statistical and clinical significance, providing for symmetry in the interpretation of results. Statistical superiority and clinical superiority are achieved, respectively, when the null and the non-inferiority margins are exceeded. We discuss a similar modification to placebo-controlled trials.

**Conclusion:**

Rules for interpretation should not favor one treatment over another. Claims of statistical or clinical superiority should depend on whether or not the null margin or the clinically relevant margin is exceeded.

## Background

Non-inferiority (NI) trials are designed to demonstrate that the experimental treatment (Test) is not unacceptably worse to an approved product (Control). By definition, Test may be slightly worse than Control while still allowing for a claim of NI. Following a finding that Test is not inferior to Control, NI trials also allow to test and conclude superiority of Test over Control. NI trials thus permit conclusions of inferiority, non-inferiority, or superiority. The criterion for concluding superiority, however, is currently asymmetric in favor of Test over Control. The goal of this article is to recommend a symmetric interpretation so that the conclusion from the trial outcome is both logically and clinically consistent. To this end, a distinction is made between outcomes that are *statistically worse* and *clinically inferior* on one hand, and that of *statistically better* and *clinically superior* on the other. Via an example, the proposed interpretation is compared with the food and Drug Administration (FDA) and European Medicines Agency (EMA) NI guidance documents and other publications. The arguments for distinctly different conclusions of statistical and clinical superiority extend also to placebo-controlled trials, to allow for different types of claims depending on the magnitude of the Test drug effect compared to placebo.

Our argument for a distinction between a statistical versus clinical superiority follows the logic articulated in [1] and several references therein. The authors discuss the role and subsequent interpretation from testing null hypotheses of equality of means. They argue that ‘When A and B are different treatments, μ_A_ and μ_B_ are certain to differ in some decimal place so that μ_A_ – μ_B_ = 0 is known in advance to be false and μ_A_ – μ_B_ ≠ 0 is known to be true’. In the context of NI and superiority trials, their argument leads to the inevitable conclusion that simply asserting that one drug is statistically superior to another (following the rejection of the null hypothesis) is a matter of proving the obvious. What is necessary for concluding clinical superiority is a statistical conclusion that one drug is better than the other by a clinically meaningful difference, *M*.

Non-inferiority trials are typically employed when, in the presence of an approved drug, it is unethical to treat patients with placebo, or because Test is expected to offer some advantage over Control (such as a lower rate of side effects or an improved dosing regimen). One of the main steps in the design of the trial is the selection of the NI margin, *M. M* is the largest amount by which Control can be better than Test with Test still considered non-inferior to Control. The choice of *M* is obviously a critical part of the study design of NI trials but that is not the focus of this article; the reader is referred to [2–13].

For concluding NI, *M* must be ruled out, whereas for concluding superiority, the null margin must be ruled out. Note that the null margin equals 0 for testing differences in parameters or 1 for testing ratios. The logical inconsistency in interpretation arises due to the distinctly different criteria used for claiming NI which is based on *M*, and superiority based on the null margin.

## An illustrative example

The PRECISION trial [14] compared the cardiovascular safety of celecoxib to the nonselective nonsteroidal anti-inflammatory drugs naproxen and ibuprofen. The primary endpoint was the first occurrence of death from cardiovascular causes, nonfatal myocardial infarction, or nonfatal stroke. The trial was event driven, and required enrollment of approximately 24,000 patients to accrue about 600 events. The original design used a non-inferiority hazard ratio (HR; celecoxib/naproxen as primary comparison) margin of *M* = 1.33. The other criteria for establishing NI are ignored (i.e., the HR point estimate not exceeding 1.12 in the intention-to-treat and the on-treatment populations) as is the comparison of celecoxib to ibuprofen. Consider the conclusions under the conventional standard of demonstrating inferiority, NI, or superiority for different *population* HRs:

- HR ≥ 1.33: Celecoxib inferior to naproxen (or naproxen superior)
- 1 ≤ HR < 1.33: Celecoxib not inferior to naproxen
- HR < 1: Celecoxib superior to naproxen

The conventional approach makes a demarcation at the NI margin for a HR >1 to enable a conclusion of NI if the HR <1.33 or a conclusion of inferiority if the HR ≥1.33. There is no demarcation for a HR <1. This leads to the following inconsistency: Celecoxib is judged non-inferior to naproxen if, say, the HR = 1.14, yet celecoxib is judged *superior* to naproxen if it is better by the same amount, i.e., by 1/1.14 = 0.88. We emphasize that this is not a semantic issue; it is a logical one. Having decided the margin, the term NI means that Test is not worse than Control by more than *M*. If Test is better than Control by an amount less than *M*, then it is illogical to confer the label ‘superior’. There is no clinical rationale to consider naproxen inferior to celecoxib if the HR = 0.88 yet consider celecoxib not inferior to naproxen if the HR = 1.14. Adding a demarcation at 1/1.33 = 0.75 would lead to the consistent interpretation that with a HR = 0.88 naproxen is concluded to be not inferior to celecoxib. The following symmetric interpretations are proposed.

- HR ≥ 1.33: Celecoxib *clinically* inferior to naproxen (or naproxen *clinically* superior)
- 1 ≤ HR < 1.33: Celecoxib *statistically* worse but *clinically* similar to naproxen
- 0.75 ≤ HR < 1: Celecoxib *statistically* better but *clinically* similar to naproxen; 0.75 = 1/1.33
- HR < 0.75: Celecoxib *clinically* superior to naproxen (or naproxen *clinically* inferior)

**Fig. 1:**
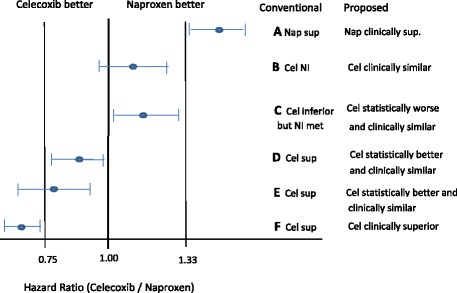
Comparison of interpretation of outcomes between the FDA NI guidance document and what is proposed. FDA interpretation is provided for scenarios C and E (Figure 2 in [2]). See also Figure 6 in [4] which discusses the same example. *Cel* celecoxib, *Nap* naproxen, *NI* non-inferiority, *sup* superior

These distinctions are now discussed in the context of 95% confidence intervals (CIs) and compared with regulatory documents and the literature. Six scenarios, A–F, are shown in Fig. 1. The FDA guidance document (Figure 2 in [2]) includes scenarios C and E; see also the EMA Points to Consider document (Figure 4 in [3]). Figure 6 in [4] discusses possible outcomes from the PRECISION trial.

NI (but not superiority) of celecoxib to naproxen is achieved for scenarios B and C. Of main interest, though, are scenarios C and D, and secondarily A and F. Of note, C and D are mirror images, yet the conventional conclusions are asymmetric. The criterion for NI is met in scenario C (even if Test is statistically inferior to Control). For D, the reference point for superiority is the null margin, leading to the inconsistent conclusion that Test is superior to Control. The two scenarios, C and D, lead to the logical oddity that two trials with mirror results end up with different conclusions. To make it concrete, suppose the PRECISION trial yielded the following HR point estimate and 95% CI: 0.87 (0.80–0.95). The conventional conclusion would be that celecoxib is superior to naproxen because the upper limit is <1. However, if the trial were sponsored by naproxen, the conclusion would be that naproxen is not inferior to celecoxib because the lower limit >0.75 = 1/1.33; or equivalently, to use 1.33 as the NI margin, write the HR as the ratio of hazard rates of naproxen and celecoxib and take reciprocals of the results. This asymmetry in interpretation is clinically illogical.

The criteria for transitioning from Test’s claim of NI by reference to *M*, and for superiority by reference to the null margin, is a view held widely. Scenarios D or E are interpreted to mean that Test is better than Control (Figure 2, case 5 in [2]; Figure 4 in [3]; Figure 6, scenario A in [4]; see also [6–9, 15, 16] for similar interpretations.) The ability to claim superiority is often made in the context of not requiring any adjustment to the type I error. While that is true, the more relevant matter is attaching a consistent interpretation to NI and superiority, regardless of which treatment is test and which is the comparator. The logical interpretation of scenario C is that celecoxib is *statistically* worse than but *clinically* similar to naproxen, whereas for scenario D it is that celecoxib is *statistically* better than but *clinically* similar to naproxen. To claim clinical superiority, the NI margin needs to be exceeded, as shown for scenarios A and F.

It might be said for scenarios D or E that a claim of superiority is justified because not only is Test statistically superior to Control, it potentially may also provide other advantages such as fewer side effects or a more convenient dosing regimen. If only NI but not clinical superiority is achieved, any benefits external to the efficacy endpoint are manifestly not a reason to allow a conclusion of superiority on that endpoint. Each purported benefit is its own domain that forms part of the risk-benefit assessment of Test to Control—that is, the comingling of benefits, conveniences, and risks needs to occur outside of the formal statistical framework.

To illustrate this, suppose for example the prevailing view is that celecoxib is expected to have a better adverse effect profile than naproxen. As a consequence, some concession is granted in the criterion for declaring superiority; celecoxib is allowed to claim superiority if the upper confidence limit is less than 1. What, if upon trial completion, celecoxib’s adverse event profile is found to be no better than naproxen’s, and scenario D is observed? Celecoxib would be judged superior to naproxen, since the prespecified condition for demonstrating superiority was achieved even though, for the patient, there would be no safety benefit. This explains why any expectations concerning safety ought not to influence the choice of the margin for efficacy. The risk/benefit evaluation would incorporate formal (i.e., efficacy) and less formal (e.g., adverse events) comparisons in arriving at a final determination. Conversely, if celecoxib’s safety profile is unequivocally better than naproxen’s, the *overall* risk/benefit assessment may well be that it is clinically superior to naproxen even under scenario D. For another example, suppose celecoxib offers a more convenient dosing regimen than naproxen. Should that affect the choice of the margin—i.e., should the margin be wider than it would be without this convenience? Our position is that it should not. That advantage too is its own domain, worthy of evaluation when making the overall risk/benefit assessment comparing celecoxib to naproxen.

### Extension to placebo-controlled superiority trials

Under the current paradigm, statistical superiority is achieved if the one-sided *p* value is <0.025 in favor of Test over placebo. The term ‘superiority’ is often used interchangeably with statistical significance and clinical significance. ICH E10 [17] contains the following: “A trial using any of the control types may demonstrate efficacy of the test treatment by showing that it is superior to the control (placebo, no treatment, and low dose of test drug, active drug).”

Using arguments in [1] that a mere rejection of the null hypothesis is simply a statement of the obvious, we can distinguish between statistical and clinical superiority in placebo-controlled studies as well. Specifically, the margin for clinical superiority of a drug versus placebo would need to be proven in order to claim clinical superiority. Obviously, estimating a drug effect compared to placebo is a process often involving more than one study. Nevertheless, once that estimate has been made, an assertion that a drug has shown a clinical superiority over placebo can and should be recognized. In the current regulatory environment, two approved drugs for the same indication will usually have very similar label claims even if the magnitude of their efficacy is substantially different. The relative effect of the two drugs will sometime be realized over a period of time, after the drugs have been marketed, perhaps with a head-to-head comparison. Until then, patients and physicians can only rely on the information included on the drug label.

## Remarks

The original intent of non-inferiority trials suggests that the hypothesis tested is one-sided, and that the statistical test should appropriately be designed as a one-sided test. In most regulated trials, the lower (or upper, as may be the case) confidence limit is set at 0.025 (although, in some rare cases, 0.05 is allowed). If the sponsor of the trial is only allowed to make a non-inferiority claim based on a 0.025 level test, then no asymmetry between Test and Control is present. The asymmetry comes up precisely because regulators do allow a superiority claim following a non-inferiority finding. Confirmatory trial designs must ensure that overall type 1 error is no more than 0.025 one-sided. The sponsor is allowed to design and test hypotheses in a manner that preserves the type 1 error. The idea of allowing sponsors to make a superiority claim following a successful non-inferiority test conforms with the requirement of type 1 error preservation, which is part of the argument to allow the superiority claim. This, however, does not address the asymmetry between Test and Control; in fact, it is partially the cause of it.

This article is confined to the rationale for making a distinction between claiming statistical and clinical superiority. We have made two points: the first is that the inherent asymmetry in NI trials stemming from the ability of Test to claim superiority over Control by achieving a null margin, while also allowing to conclude non-inferiority upon achieving the NI margin, can be rectified by making a distinction between statistical and clinical superiority. For trials to be conducted in the future, the symmetric approach to inferring efficacy ought to be incorporated into the design. For trials that have been completed, the symmetric interpretation may be retroactively applied in re-evaluating clinical superiority of experimental treatment to control. The second point is that the conclusion of statistical superiority following the rejection of the null hypothesis should be considered as achieving a much lower bar than achieving clinical superiority, especially in placebo-controlled trials. If demonstrating clinical superiority in NI or placebo-controlled trials is an important objective (as opposed to just demonstrating statistical superiority), then the size of the trial will increase. Trial designers will therefore have to explicitly plan for a larger trial or make other material changes to the design, such as identifying enriched patient populations for enrollment into the trial. We recognize that, in practice, other factors such as the fraction of data that are missing, the robustness of results across other endpoints, the information from other studies, etc. are part of the evaluation of a drug effect. Nevertheless, the points made above can be incorporated into this final determination.

## Abbreviations

CI: Confidence interval
EMA: European Medicines Agency
FDA: Food and Drug Administration
HR: Hazard ratio
NI: Non-inferiority
